# The role of primary care in the cancer care continuum: a qualitative study of cancer survivors’ experiences

**DOI:** 10.1080/02813432.2022.2145848

**Published:** 2022-11-15

**Authors:** Lars Garpenhag, Anders Halling, Anna-Maria Larsson, Susanna Calling

**Affiliations:** a Center for Primary Health Care Research, Department of Clinical Sciences Malmö, Lund University, Lund, Sweden; b Regional Cancer Center South, Lund, Sweden; c Division of Oncology, Department of Clinical Sciences Lund, Lund University, Lund, Sweden

**Keywords:** Primary care, cancer survivors, continuum of care, qualitative research, focus groups, health services accessibility, Sweden

## Abstract

**Objective:**

To explore how cancer survivors have experienced their contacts with primary care after being diagnosed with cancer, focusing on the integration between cancer specialist and primary care, and participants’ views on what could make primary care services better at catering to the needs of cancer survivors.

**Design:**

A qualitative study in which data was collected through semi-structured digital focus group interviews and analyzed using a template analysis approach.

**Setting and subjects:**

Adult residents of Skåne, Sweden, who had been diagnosed with and initiated treatment for either of five common cancer forms, recruited through patient advocacy groups.

**Main outcome measures:**

A qualitative description of participants’ experiences and perceptions as expressed in focus group interview data.

**Results:**

Most participants felt that primary care services had not played a significant role for them, despite patterns of both increased and unmet health needs. Insufficient coordination and communication with specialist cancer care, low availability, lacking personal continuity, low cancer competence and lacking commitment to cancer-related needs were presented as barriers to satisfactory primary care. A strengthened bond between cancer and primary care services, privileged access, and holistic perspectives were all suggested as measures to make primary care more suitable to cancer survivors’ needs.

**Conclusion:**

The study suggests that cancer survivors experience a range of issues that hinders primary care services from playing a productive role in the cancer care process. The results speak for a need for interventions to remove barriers to satisfactory primary care contacts in this group of patients.

**KEY POINTS**
- The growing number of cancer survivors highlights the role of primary care services in the cancer care continuum.
- Despite the presence of unmet needs, few cancer survivors felt that primary care services had been significant to their care.
- Survivors identified a number of barriers to satisfactory primary care, including lacking coordination and communication between cancer and primary care.
- Strengthened links between healthcare services, privileged access, and holistic perspectives were suggested to improve primary care delivery for cancer survivors.

## Introduction

Globally, the number of persons who are diagnosed with cancer is on the rise, and is expected to continue increase in the foreseeable future [1]. Aging populations, together with the development of methods and technologies that allow earlier detection and more efficient treatment of many forms of cancer, also mean that an increasing number of people will live on after primary cancer treatment, with or without chronic disease [2]. In Sweden, according to projections, the number of people living within 10 years of receiving a cancer diagnosis will more than double until the year 2040 – as will the societal costs associated with cancer [3].

The growing number of cancer survivors – living with or beyond cancer [4] – presents a challenge to healthcare services, since cancer survivorship in itself is associated with complex and often major health needs. Physical as well as psychological, social and existential needs can arise both during active treatment and afterwards [5]. In addition, non-cancer related comorbidities and multimorbidity – a problem especially in the large sub-group of older adult cancer survivors – run the risk of being overshadowed by health needs directly connected to the cancer [6].

The acknowledgement of cancer survivors’ needs, and the resulting strain on healthcare resources, has stimulated an interest in the role that primary care services currently play in the cancer care continuum, as well as in how this role could be developed to contain costs and increase the quality of care. Aside from the prevention and detection of cancer that regularly fall within the purview of primary care responsibilities [7], and efforts to provide cancer survivors with primary care services in general [8], studies have assessed primary care involvement in the provision of basic forms of cancer follow-up, rehabilitation and supportive care [9–11]. Meanwhile, primary care professionals’ lack of cancer-specific competence, and deficient communication and coordination between cancer specialists and primary care, have proven to be major stumble blocks to the integration of primary care services into the cancer care continuum [7,12–14]. Attempts to over-bridge the divide between cancer specialist and primary care have involved both the development of organizational models for follow-up care [15–16] and the implementation of survivorship care plans to help planning and information transfer [17].

In Sweden, the increased scholarly interest in the needs of cancer survivors has generated a substantial body of studies on health outcomes, health needs and quality of life in specific sub-populations of cancer patients [18–19], and on survivors’ access to supportive care [20–21]. Sweden has a comprehensive public primary care system organized on the regional level by the county councils. Services are covered by the mainly tax-financed universal health insurance, but delivered by a mix of public and private providers [22]. The importance of primary care involvement in the provision of good quality healthcare to cancer survivors is acknowledged in current policy documents [23]. However, with the exception for an inventory report on available cancer rehabilitation resources in primary care in Stockholm [24], neither Swedish primary care services’ actual nor their potential roles in this context have been systematically studied. While much can be learned from studies on the subject that have been conducted abroad, the vast international differences in health care organization emphasize the need to study different national contexts.

The aim of this qualitative study was to explore how cancer survivors have experienced their contacts with primary care services after being diagnosed with cancer, with special focus on the integration between cancer specialist and primary care, as well as their views on what could make primary care services better at catering to the needs of cancer survivors. In order to provide important context for participants’ experiences, we also explored their self-assessed health needs.

## Materials and methods

Data for this qualitative descriptive study was collected through semi-structured digital focus group interviews held in the fall and winter of 2021 and early 2022, and analyzed using a thematic approach. The study was approved by the Swedish Ethics Board (file no. 2021-03534).

### Data collection

The conduction of focus groups is an established technique for collecting data in health studies concerning patients’ experiences of illness, healthcare and related phenomena, and has been described as a particularly efficient way to gather exploratory data [25]. The choice to conduct the interviews digitally was based on considerations caused by the ongoing Covid-19 pandemic. Assessments have suggested that in comparison to conventional physical focus group meetings, those conducted using videoconference technology display similar dynamics of interaction between participants [26], and yield similar data [27]. In addition, it has been proposed that digital interviews facilitate the participation of persons who might otherwise be hindered by poor health [28].

Inclusion criteria for participation were adult age (age 18 or over) and self-reported initiated primary treatment for breast cancer, prostate cancer, lung cancer, colorectal cancer or malignant melanoma. Terminal phase cancer patients were excluded, as were non-fluent Swedish speakers. Initially, participants were sought among residents in the cities of Malmö and Lund and adjacent municipalities, but due to a modest turn-out the area of recruitment was widened to the whole Skåne region (Sweden). Participants were recruited with the help of six patient advocacy groups (PAGs) that organize people with the included diagnoses. The PAGs all received the same advertisement to distribute digitally to their members. The channels of distribution included digital mailing lists, social media posts and announcements on PAG web pages.

Prospective participants announced their interest *via* e-mail, upon which they were sent an information package by mail, and were asked to complete forms asking for basic personal information and written informed consent to participation in the study. In the end, no selection had to be done among those who completed both forms. Information was sent out beforehand about how to prepare for a digital interview, and about what hard- and software was needed. To facilitate a comfortable discussion in the online setting, the size of the interview groups was kept relatively small, aiming at including four to six participants in each session [25].

The interviews were carried out using Zoom Meetings (Zoom Video Communications, Inc.). A moderator (first author) conducted the interviews assisted by an observer (the other authors on rotation) who took notes, asked occasional follow-up questions and stood by to help participants with technical issues. The interviews were based on a questioning route, containing a number of open-ended questions prepared beforehand based on the study aims (Supplementary material). The semi-structured character of interviews made data collection flexible, facilitating exploration of experiences and views that were not expected at the outset of the study. The audio was recorded locally on a computer, and transcribed verbatim by an external resource.

### Analysis

The analysis aimed at creating a qualitative description of relevant parts of the data, keeping close to the actual language and concepts of the study participants without the addition of any more elaborate elements of theoretical interpretation [29]. The analytic work was guided by a template analysis approach. Template analysis is a systematic but adaptable approach to thematic analysis in which a coding template is created and used as a tool to categorize data and hierarchically organize it to help analysis [30].

The coding was initiated by the first and last authors, who independently created coding templates based on a subset of the data, starting out with a small number of à priori high-order codes reflecting the research interests of the study. The drafted templates were then discussed and merged into a proposal that was discussed with the rest of the authors. A modified version of the coding template was then applied to the whole dataset by the first author, who identified major themes and continuously made minor modifications to the structure as need arose. After that, a revised proposal was presented to the project group for discussion before analysis was completed. An illustration of the analytic procedure is found in Table 1 (the final template can be obtained by request). While the data was in Swedish, the coding and analysis were done in English from the start. The quotes in the article were translated from Swedish and edited for clarity by the first author. To protect their identities, participants’ names have been changed to fictitious ones.

**Table 1.** Example of the analytic procedure.

Quotes	Code	Sub-theme	Theme	Overarching theme
What comes to mind is that… when you’re through with [the primary cancer] treatment and don’t belong, so to speak… I belonged to the surgery department in [City], and then you’re supposed to go to the primary care center, and there, I feel, I haven’t really been picked up.	Transferred into a void	–	A fragmented healthcare process	Survivor experiences of primary care
Primary care services, in my world, have not had any role at all, and it hasn’t… It hasn’t been an issue at all for some reason. They don’t have the … they don’t have either an interest or conditions to do anything. Completely uninterested.	The quantitative role of primary care	–	Primary care utilization and satisfaction	Survivor experiences of primary care
	Commitment	Low commitment	Barriers inherent to the organization of primary care	Survivor experiences of primary care
No, not… well it is always good to get hold of someone you can talk to, if you’d like that, but that doesn’t exist today, because… it just doesn’t exist. You get to call during telephone hours, and if they have the time to take you on they do, and otherwise they don’t.	Availability	Poor availability	Barriers inherent to the organization of primary care	Survivor experiences of primary care

## Results

After a brief description of participants’ characteristics, we describe the results from the thematic analysis. To a great degree, the interviews came to dwell on negative experiences of the healthcare system in general and primary care in particular, and the analysis reflects this general pattern. It is important to note, however, that this does not necessarily mean that all the presented negative experiences were shared by all participants. The first theme revolves around *participants’ health needs*. Then we go on to present three themes pertinent to *survivor experiences of primary care*. The first, *primary care utilization and satisfaction*, summarizes how participants described their use of primary care services. The second, *a fragmented healthcare process*, describes experienced deficiencies in the relationship between hospital specialist and primary care services. The third, *barriers inherent to the organization of primary care*, describes a number of problems that participants felt had hindered them from satisfactory primary care utilization, presented as four sub-themes: *poor availability*, *lacking personal continuity*, *lacking competence* and *low commitment*.

### The participants

A total of 20 participants (11 men, 9 women) were interviewed in 5 groups of 3–5 persons each. The groups were mixed in regard to gender and diagnosis, and the interviews lasted approximately 50–75 min. The age span among the participants was 48–78 years (mean = 65.2 years). Eight of them had been diagnosed with colorectal cancer, 5 with prostate cancer, 3 with breast cancer and malignant melanoma respectively, and 1 with lung cancer. The approximate time since diagnosis varied, from less than a year up to 10 years. A little over half of the participants received their diagnoses 3 years ago or more, 6 of them 5 years ago or more. The sample included both patients still in primary treatment and those who had finished it. Three participants were undergoing treatment for chronic cancers.

### Participants’ health needs

The great majority of participants had experienced an increased general need of healthcare after they had been diagnosed with cancer, with only three persons claiming that their needs had remained more or less unchanged. Two of the latter had undergone uncomplicated and successful treatments, while the last one was still at a relatively early stage of treatment. Most had experienced novel physical needs, while around half reported having psychological needs that they associated with their illness, some of which had appeared only after finished treatment. The participants touched upon, but had much less to say about, needs of an existential or social character. Several of them emphasized that cancer had been a shared burden for them and their significant others, and that the needs of family (including children) should not be forgotten by healthcare actors.

Over half of the participants complained about health needs that had not been met by healthcare, or that had eventually been met, but only after lots of effort on the part of the patient him- or herself. These needs were associated with considerable suffering that participants felt should actually be avoidable. While the unmet needs were diverse in character, two patterns are discernable. First, while routine physical needs tended to be met, healthcare services seemed to be less successful in handling physical needs and symptoms of a rare or unexpected nature.

I had lots of problems with lymphedema in my legs. […] and well, I work a lot with that myself, and then they told me that that they didn’t… they had more experience in dealing with breast cancer, you know, arm edemas.

*Lina (melanoma)*

The second pattern concerned unmet psychological needs. Most who had experienced such needs had felt well taken care of during primary treatment, with access to counseling at the treating specialist clinics. However, after leaving treatment, several participants hadn’t been able to – or even known that they could – procure such services from their primary care centers. A few had felt forced to seek psychological help privately, since they felt that the counseling that had been offered by the public healthcare system did not fill their needs.

*Moderator*: So, you experienced some kind of mental backlash?

*Jonas (colorectal cancer)*: Yes, absolutely.

*Moderator*: That you would’ve needed help to cope with?

*Jonas*: Yes, and somehow I’ve always… perhaps not believed, but… that everyone else has received [help], but that I’ve missed out on it – that there exists this holy cancer help center where everyone ends up, but perhaps that’s not the case.

### Survivor experiences of primary care

#### Primary care utilization and satisfaction

The extent of participants’ utilization of primary care varied from person to person, as did the significance they attributed to it. For many of them, however, it had played a marginal role in their lives since they had received their cancer diagnoses. These participants – including persons both in and out of primary treatment – claimed that their contacts with primary care had been minimal or even non-existent. Basically, it had been limited to, at most, routine follow-up matters, e.g. blood work and the maintenance of PICC-lines. A few others had clearly had more contacts with their primary care centers, for instance in search for help with what they believed to be side-effects of cancer treatment, or for routine checkups for other chronic diseases, e.g. diabetes mellitus and hypertension. Yet, primary care contacts had played a significant role only for a small number of participants.

I haven’t sought contact. [The primary care center] sent the first referral to the hospital to initiate the further investigation, through which I eventually got my diagnosis, but I haven’t had any reason to be in contact with them.

*Börje (prostate cancer)*

I’m actually seeing a primary care physician tomorrow, whom I don’t know at all. And I haven’t been there since before I… or when I got my cancer diagnosis.

*Lisa (colorectal cancer)*

Participants’ satisfaction with their primary care relationships varied as well. Limited contacts did not in themselves imply dissatisfaction. Some participants argued that they had simply not seen any reason to seek primary care, or that they were more or less satisfied overall, even with limited interactions.

Well, I haven’t had… I have gone there to get some shots and it has worked out great.

*Frank (colorectal cancer)*

However, as will become evident in the upcoming sections, the general impression of the interviews was that many participants had experienced their contacts with primary care as frustrating to various degrees.

#### A fragmented healthcare process

A non-functioning relationship between the primary care centers and hospital-based cancer specialist care appeared as a major perceived barrier to satisfying primary care contacts. Participants had experienced deficiencies in the coordination between providers at key points in the care process, as well as a continuous absence of communication between providers from the involved specialties.

Participants identified a lack of coordination in the interface between hospital-based cancer treatment and primary care as a huge source of frustration. Most who had an opinion seemed satisfied with the initial transition from primary to hospital specialist care at the time when the cancer was detected. The integration between services during and after treatment, in contrast, had turned out to be more problematic. While a majority of participants had had some kind of long-term plan laid out for them early on during cancer treatment, these plans had generally been centered on treatment and therapeutic follow-up. No participant was aware of any primary care professionals ever being involved in making the plans, and neither did their plans include anything about the role of primary care. In some cases, planning did not at all cover what could be expected after finished treatment.

*Robert (colorectal cancer)*: I haven’t seen any… any personal care plan.

*Moderator*: Not at all?

*Robert*: No. But I’ve read the national care guidelines.

*Moderator*: Yes, so you know what’s *supposed* to happen?

*Robert*: Yes, but it doesn’t seem like those who provided my care ever read it.

Only one participant reported having been aware of an active hand-over to primary care successfully taking place. Consequently, many of those who had finished treatment had found themselves in a gap afterwards. They were no longer supposed to contact the hospital unit where they had been treated – a change that could be experienced as drastic in itself – and they had not been introduced to any contacts that would supplant the old ones either. As a result, participants had felt like they had been abandoned in a void, confused over who was responsible for their care. The transition from being a ‘cancer patient’ back to being a ‘regular patient’ had been imbued with a sense that it was up to the patient him- or herself to make things work. To some, this prospect appeared daunting.

It feels like they are really good at saving your life, but then… what comes afterwards is difficult as well you know, and then you have to find your own way, the best you can.

*Lina (melanoma)*

In addition, according to participants, such instants of poor coordination went hand in hand with a lack of ongoing communication and information sharing between the different branches of healthcare. Some used drastic terms like ‘separate worlds’ and ‘watertight compartments’ to describe the relationship between hospital and primary care specialists. One recurring complaint was that professionals whom participants had encountered in primary care had been ignorant of their cancer history. This was commonly attributed to the fact that hospitals and primary care centers in Region Skåne run two separate record systems. While some primary care professionals have access to hospital records, this is not by default. In addition, accessing the system requires both extra effort and active patient consent. Some participants also turned the information deficit into a question of time and resources, arguing that you could not expect a primary care physician to have the time to read up properly on the oftentimes extensive records of a cancer survivor.

There is some kind of system error in all of this, you know. […] Then… I can’t comprehend why there is no connection. Because as it works today, many providers don’t even use the same computer system.

*Peter (colorectal cancer)*

Meanwhile, great hopes were vested in a future implementation of survivorship care plans as a means to facilitate inter-specialist communication and the coordination of care. The ongoing but yet to be finished introduction of a unified digital records system for all public healthcare in the region was also believed to bear a potential for more efficient information transfer between providers. It was suggested that if only providers could work in greater coordination, primary care had the potential to fill an important role, and even serve as coordinating node, in survivorship care.

Actually, my cancer treatment is finished now, even if I’ll attend check-ups every year […]. So, the hospital doesn’t… At the stage where I am, they don’t really have any reason to actively refer me to primary care, because I’m done. But I think that a lot of the rehabilitation that you might need both during and after a cancer diagnosis could have been handled by primary care, if healthcare would have been good at hand overs. Yes, I believe so.

*Karin (breast cancer)*

#### Barriers inherent to the organization of primary care

Besides factors pertaining to the relationship between services, participants had experienced a wide range of problems that they associated with the organization of primary care itself. The flaws identified could be summarized as poor availability, lacking personal continuity, lacking competence and low level of commitment.

##### Poor availability

Several participants complained that they had not been able to secure the primary care resources they needed, when they needed them, and that long waiting times made it difficult to get appointments in a timely manner. One participant claimed to have successfully increased her access to primary care through playing what she referred to as ‘the cancer card’ – consciously using her cancer diagnosis to be allocated greater resources – but this did not seem to be a universally successful strategy. Adding to the problems, several participants lamented, was that even if you manage to see a professional, there is never enough time to deal with all of your needs. Thus, having an ongoing contact, e.g. because of other chronic disease, was not in itself a guarantee that your needs would be met. Some had found it complicated to even get in contact with their primary care center. One issue in particular was what participants found to be a low level of digitalization and poor IT solutions that made communication between patients and professionals difficult.

Looking ahead I have this fear that if a birthmark starts to itch, will I need to wait for six months again before I can get to the right place? Because that was the time it took the last time counting from the date when I contacted my primary care center.

*Rut (melanoma)*

And just to give an example from my primary care center. If I attend a yearly check-up [for non-cancer related chronic illness], and want to bring up something else. No, I can’t do that, because you’re only allowed to talk about one disease at a time.

*Olof (prostate cancer)*

The implementation of privileged fast-track access to primary care for cancer survivors was suggested as one solution to problems with availability. The formation of dedicated cancer teams in primary care was proposed as one way to achieve this, and to facilitate access to resources such as, for instance, psychotherapy. In the same spirit, there were wishes that primary care providers should be more proactive, and initiate contact with patients immediately upon their discharge from cancer treatment.

There should’ve been a team who could pick you up when you’ve got your diagnosis, and when I come to the primary care center I shouldn’t need to tell them what I suffer from. It is very much a matter of [having stamina], to be able to seek help yourself […] and it isn’t always that easy to be that strong, so…

*Berit (lung cancer)*

##### Lacking personal continuity

Another recurring criticism considered personal continuity. In contrast to ideals about having an ongoing relationship with a set primary care team, participants had regularly met new faces, and in some extreme cases not even met the same professionals twice. Together with the problems associated with the transfer of information, this led to awkward situations in which they had to retell their medical histories over and over when visiting the primary care center. In addition, some felt that it made the encounters with professionals feel less comfortable, and affected the quality of care. Even what should be mere routine tasks – like the cleaning of PICC-lines – could feel precarious if the staff performing them never got the chance to grow accustomed to the procedures.

Well, primary care… If we say that the urologist scores a five on a five-grade scale […] primary care scores a plain one, because you never get to see the same physician twice. The last, I would say, five to six years I have seen different physicians at every new visit.

*Olof (prostate cancer)*

Several participants yearned for professionals to employ a more holistic approach that would entail a more coherent management of their needs, and the maintenance of stable and continuous relationships between patients and practitioners was in turn described as a prerequisite for this to work. Not only would it make interactions more trustful, but it would also enable the production of personal knowledge needed for a truly holistic healthcare encounter.

If you’ve got a diagnosis like cancer, in my case – and I also suffer from hypertension and diabetes that make me go to annual check-ups – then shouldn’t they put more focus on… you know, ‘how are you?’ – a chat you know, […] and there you could follow-up on the whole human being. Because, I shouldn’t have to see specialists and go to the urologist in [the city] for more basic things, should I? Primary care has to be able to manage such stuff somehow.

*Olof (prostate cancer)*

##### Lacking competence

Participants also shared anecdotes that served to show a lack of confidence in primary care professionals’ competence in matters relating to cancer. Experiences included physicians who could not interpret symptoms correctly, dieticians who were unable to give advice and nurses who did not know how to properly administer endocrine therapy injections. In addition, examples were brought up of what could be termed structural or organizational incompetence, for instance that primary care centers had been unable to carry out certain testing, or didn’t have routines in place to manage follow-up care processes, forcing patients to turn elsewhere.

The general norm in this country is that primary care dieticians… when it comes to nutrition for instance, when you suffer from short bowel syndrome and the like, they can’t handle it. They are very happy to see us, but they can’t give us anything. On the contrary, we patients can teach them quite a lot.

*John (colorectal cancer)*

##### Low commitment

Finally, there was a notion that primary care professionals simply aren’t very committed to the specific needs of cancer survivors. Several participants felt that their problems hadn’t been taken seriously, and some described what they had found to be a resistance, a ‘conservative attitude’, or even attempts to dodge responsibility. In the case of one female participant, this had resulted in her ‘being passed back and forth between […] primary care center and the oncologist’. Meanwhile, participants in one of the interview groups saw fully rational reasons behind the lacking commitment, arguing that primary care centers have enough on their plate already, and that they suffer a lack of financial incitements to be more committed.

It is so very frustrating, they don’t offer me anything, or move forward with anything, so I have to be the one suggesting, for instance, a new medication, or ask them for help to contact [other providers], but they don’t… They haven’t examined me or referred me anywhere.

*Cecilia (breast cancer)*

## Discussion

Despite a general pattern of increased health needs – and the presence of unmet physical and psychological needs – primary care services had played a significant role only for few of the cancer survivors who were interviewed in this study. This is surprising for several reasons. First, many of the participants were of ages that are clinically associated with the onset of chronic disease and increased utilization of primary care. Second, previous studies on cancer survivors have both shown a clear increase in primary care utilization post-diagnosis [11], and identified general practitioners as having an important informal role in survivorship care [31]. Although participants mentioned other chronic diseases like hypertension and diabetes, the treatment of these seemed of less importance than that of the cancer related needs. We made inquiries about primary care utilization in general as well, but it is possible that, due to the situation, the participants were more focused on the cancer needs, leaving this subject insufficiently covered.

Among the participants, there was wide-spread discontent with certain aspects of how primary care services work, and with their lack of coordination and communication with services specialized in cancer care. The absence of a link between providers created uncertainty about the delineation of professional responsibility, but also shifted responsibility to the patient him- or herself to take on an active role. In addition, low availability, broken continuity, low cancer competence and lacking professional commitment to work with cancer related needs were all factors that participants felt had made it difficult to access satisfactory primary care. To a great degree, these findings harmonize with those in previous studies, suggesting that the integration of primary care into the cancer care continuum in Sweden is associated with similar challenges as in many other countries, regardless of differences in the organization of healthcare provision. Gaps in the transition between different phases of cancer care have been experienced by patients elsewhere as well [6,15], as have deficiencies in inter-specialist communication [12–14,16]. Other barriers identified in our study – like low levels of availability [17], and a concern over lack of training in cancer related issues among primary care professionals [16] – have been acknowledged abroad as well.

In the last years the Swedish government has proposed a major shift in the healthcare organization with a greater focus on primary care and offering proximity to patients [32]. Our results indicate that the needs of cancer patients are currently insufficiently met in primary care and suggest a stronger focus on this patient group from decision-makers. Some of the reported issues, notably perceived problems with availability and personal continuity in primary care, are far from unique to this particular group of patients [33]. Quantitative evidence is needed in order to see if these issues affect cancer survivors to an extent that set them apart from other populations.

The variety of problems both internal and external to the organization of primary care that were brought up during the interviews was mirrored in the forward-looking views that participants presented about how primary care services could be made more suitable to caring for cancer survivors. Several of the discussed improvements chime strikingly well with current healthcare guidelines, and bear similarities to reforms that are already underway. For instance, an enhancement of the links between specialist cancer care and primary care services, e.g. through the active use of care plans and active referrals, are supported by current national guidelines for cancer rehabilitation [23]. Wishes for improved personal continuity, and the implementation of holistic perspectives in the primary care encounter, meanwhile, resonate well with ongoing efforts to promote person-centered care [34]. Suggestions concerning the creation of special functions for cancer survivors in primary care have been promoted by PAGs and suggested in a recent report from another region in Sweden [24]. While it is easy to see how it could increase cancer survivors’ access to primary care, it is clear that the implementation of such measures would require a thorough assessment of costs and benefits that place the needs of cancer survivors in a greater context of primary care patient need.

A limitation in this study is that the choice to recruit through PAGs might have led to selection bias, as it can be expected to have given priority to persons who are more engaged in healthcare policy issues than the average cancer survivor, and who possess both health and other means that make it possible for them to speak for themselves. One can speculate that this led to the unintended exclusion of persons with low socioeconomic status and – together with the language requirement – immigrant backgrounds. Thus, while the analysis is valid in the sense that it represents a range of experiences and views held by cancer survivors, one cannot rule out that there are perspectives that are not covered.

## Conclusions

The study suggests that cancer survivors in the studied region experience a diverse range of issues that they feel hamper their contacts with primary care providers, and hinders such services from playing a productive role in the cancer care process. On a policy level, this speaks for a need for interventions to remove barriers to satisfactory primary care contacts in this group of patients. However, further studies are needed to quantitatively assess the scope of these perceived problems, and not least, the extent and distribution of negative experiences among cancer survivors.

## Supplementary Material

Supplemental Material
