# Supplementary material for: The role of primary care in the cancer care continuum: a qualitative study of cancer survivors’ experiences
Source: Scand J Prim Health Care. 2022 Nov 15;41(1):13–22. doi: 10.1080/02813432.2022.2145848 (PMC10088974; doi:10.1080/02813432.2022.2145848)
Supplement: Supplemental Material [file IPRI_A_2145848_SM6275.docx]

Questioning route

(Translated from the original Swedish version by the first author)

1. First, we want each and every one of you to tell us your first name and your age. We would also like to know how long it has been since you received your most recent cancer diagnosis.
2. How are you all doing today?
3. Apart from the treatment of the cancer itself, how do you feel that your healthcare related needs have changed since you were diagnosed with cancer?
4. Have you experienced problems that you feel have not been addressed? Do you have needs that have not been acknowledged or met by a healthcare provider?
5. What kind of interactions have you had with primary care services since you were diagnosed with cancer? And how do you feel that these interactions have worked?
6. Would you have liked to have more interaction with primary care services, at the present time or earlier on during your cancer trajectory? If so, why?
7. How do you feel that the communication has worked between the professionals who have been responsible for your cancer treatment and professionals in primary care?
8. Since you received your cancer diagnoses, have you felt safe in your knowledge of which services are responsible for which of your healthcare needs?
9. Is there anything that you feel that we have missed talking about, concerning the role of primary care services in your care, or needs that are not met?
